# Sex Differences in 90-Day Functional Outcomes for Acute Ischemic Stroke Patients With Obstructive Sleep Apnea

**DOI:** 10.7759/cureus.39652

**Published:** 2023-05-29

**Authors:** Adriana N Devenish, Tariq Nisar, Amber Criswell, David McCane, Dinh Thao, Ken Chyuan Ling, David Chiu, Rajan Gadhia

**Affiliations:** 1 Neurological Institute, Houston Methodist Hospital, Houston, USA; 2 Neurology, Houston Methodist Hospital, Houston, USA; 3 Neurology, Houston Methodist Neurological Institute, Houston, USA

**Keywords:** modified rankin scale (mrs), hypertension, atrial fibrillation, atherosclerosis (as), brain hypoxia, ais (acute ischemic stroke), obstructive sleep apnea (osa)

## Abstract

Background

Obstructive sleep apnea (OSA) is characterized by repeated episodes of either full or partial obstruction of the upper airway. OSA is an independent risk factor for acute ischemic stroke (AIS) and a contributor to other key risk factors. OSA may damage endothelial and brain tissues and worsen outcomes following AIS. We aimed to evaluate the impact of sex differences on 90-day functional outcomes following AIS in an OSA population, as measured by the modified Rankin Scale (mRS) score.

Methodology

We performed a retrospective study of patients with OSA and AIS from the Houston Methodist Hospital Outcomes-Based Prospective Endpoints in Stroke (HOPES) Registry from 2016 to 2022. Patients with charts that noted a diagnosis of OSA before AIS or within the 90 days following AIS were included. A multivariable logistic regression model was constructed adjusting for demographics, first admit National Institutes of Health Stroke Scale (NIHSS), and comorbidities on the binary outcome. The odds ratios (ORs) and 95% confidence intervals (CIs) were reported, providing likelihood estimates of a shift to higher mRS for a given comparison between females (reference category) and males. Statistical significance was defined as two-tailed p-values <0.05 for all tests.

Results

From the HOPES registry, 291 females and 449 males were found to have OSA. Males had a higher proportion of comorbid conditions such as atrial fibrillation (15% vs. 9%, p = 0.014) and intracranial hemorrhage compared to females (6% vs. 2%, p = 0.020). The multivariate logistic regression model showed that males were at two times higher risk for developing poor functional outcomes at 90 days (OR = 2.35, 95% CI = 1.06-5.19), p < 0.001).

Conclusions

Males were found to have two times higher risk for developing poor functional outcomes at 90 days. This may be due to more severe oxygen desaturation, increased susceptibility to oxidative stress, and greater frequency of full airway obstruction in males. Greater emphasis on early diagnosis and treatment of OSA may be necessary to reduce the disproportionate incidence of poor functional outcomes, particularly among apneic male stroke survivors.

## Introduction

Obstructive sleep apnea (OSA) is characterized by short and repeated episodes of either full (apnea) or partial (hypopnea) obstruction of the upper airway. This episodic interruption of breathing translates into intermittent hypoxia and repeated arousals from sleep. Sleep apnea is an independent risk factor for acute ischemic stroke (AIS) and a contributor to the development and treatment resistance of other risk factors, primarily hypertension and atrial fibrillation [1-3]. OSA may also hinder healing, damage endothelial and brain tissue, and worsen outcomes following ischemic stroke by various mechanisms. Insufficient flow of oxygen to the brain during hypoxic episodes may slow or prevent the healing of cerebral ischemic injury and trigger neuron death [4]. Furthermore, the disruption of sleep may reduce brain tissue repair and increase the risk of post-stroke depression, which often portends poor outcomes [5-7].

Elevated blood pressure after apneic arousals may escalate to sustained hypertension or greater blood pressure variability [8,9], factors that are associated with poor outcomes following AIS [10,11]. Acute hypoxia stimulates oxygen-sensitive chemoreceptors, activating increased sympathetic activity [12,13], and chronic intermittent hypoxia (CIH) elevates the secretion of endothelin, a powerful vasoconstrictor and baroreceptor inhibitor. The peripheral vasoconstriction and accelerated heart rate induced by sympathetic hyperactivity and baroreceptor inhibition contribute to elevated blood pressure. CIH may eventually lead to increased carotid body sensitivity to hypoxia and a gradual increase in baseline carotid body sympathetic activity. It has been hypothesized that this enhancement of the carotid body chemoreflex is responsible for sustained sympathetic hyperactivity during the daytime in OSA patients [13].

CIH also triggers decreased levels of nitric oxide (an endothelium-derived relaxing factor), resulting in endothelial dysfunction and increased vasoconstriction. OSA patients exhibit impaired endothelial repair capacity [14]. Endothelial dysfunction may impair perfusion to the brain, exacerbate hypertension, and contribute to increased stroke risk.

Rodent and cell culture models of CIH offer insights into the molecular mechanism by which OSA may enhance chemoreflex, provoke endothelial dysfunction, and induce oxidative stress. In these models, CIH increases hypoxia-inducible factor-1α (HIF-1α) expression, corresponding to increased transcription of pro-oxidant enzymes, and decreases hypoxia-inducible factor-2α (HIF-2α) expression, corresponding to decreased transcription of antioxidant enzymes. The result is a rise in reactive oxygen species (ROS) and oxidative stress that damages cells and contributes to further sympathetic hyperactivation, hypertension, and endothelial dysfunction. The increase in endothelin due to OSA may be the result of increased ROS because increased endothelin-1 (ET-1) levels in rodents exposed to CIH were due to ROS-dependent activation of the enzyme that generates active ET-1 [12,13]. Lipid-rich brain tissue becomes an easy target for ROS and oxidative damage. Significant cerebral atrophy has been observed in patients with moderate-to-severe OSA and is likely the result of both oxidative stress and tissue damage caused directly by hypoxia [15]. Oxidative stress biomarkers can be used as predictors for functional outcomes after AIS [16]. Oxidative stress is also a key causative factor of cerebral small-vessel disease (SVD) [17]. Endothelial cells are damaged not only by apnea-induced oxidative stress but also by a phenomenon known as non-dipping. Non-dipping involves a blood pressure drop of less than 10% during sleep and is associated with SVD, a higher risk of recurrent stroke, and poor outcomes after a stroke [18].

Inflammation from OSA-related cell damage triggers the secretion of proinflammatory cytokines such as tumor necrosis factor-α and interleukin-6 [19]. Elevated secretion of these proinflammatory molecules may aggravate neurodegeneration, atherosclerotic lesion progression, and brain tissue reperfusion injury [20]. Elevated tumor necrosis factor-α correlates to poorer outcomes following AIS [21].

The obstructed attempts at forced inhalation during apneic episodes may create negative intrathoracic pressure. Negative intrathoracic pressure swings put stress on the atrial walls and may result in atrial remodeling that predisposes to arrhythmia and new-onset atrial fibrillation. Additionally, the sympathetic overstimulation, oxidative stress, and systemic inflammation fostered by OSA further elevate susceptibility to atrial fibrillation and poor treatment outcomes [22-24].

While OSA has been well-established as a risk factor for poor functional outcomes following AIS [1], the impact of sex on the strength of association between OSA and functional outcomes remains unknown. OSA has long been considered a predominantly male disease and is more common in men [25], but this gap could be in part attributed to underdiagnosis in women. Women tend to report more *atypical* symptoms such as fatigue, insomnia, mood disturbances, and depression and are less likely to report snoring or witnessed apneas [26]. Men present with more severe OSA in terms of Apnea Hypopnea Index (AHI) and oxygen desaturation [27,28].

However, the gap in OSA prevalence and severity between men and women narrows with age. Women report greater impairment in quality of life than males with similar AHI, and OSA symptoms manifest at lower AHI in women, indicating that AHI is not always an adequate measure of clinical OSA severity for women [29,30]. Women also tend to experience shorter apneic episodes than men, a higher proportion of hypopneas, and greater clustering of episodes during rapid eye movement (REM) sleep (apneic episodes in men occur largely during non-rapid eye movement sleep (NREM)) [31]. The greater prevalence and AHI severity of OSA in men are likely due in part to sex differences in upper airway length. Men have proportionately longer upper airways which is linked with greater collapsibility of the upper airway and greater OSA severity [32,33]. Sex differences in fat distribution also contribute to greater upper airway collapsibility in men. Men exhibit more centrally distributed fat, while premenopausal women exhibit a more peripheral pattern of fat distribution with less fat deposited around the upper airways and neck [34]. Men and women also display differences in ventilation during sleep. Ventilatory demand during NREM sleep and ventilatory response during arousal is typically greater in men [35,36]. Furthermore, compensatory responses to upper airway obstruction during NREM sleep were found to be more effective in obese apneic females than in obese apneic males [37]. Progesterone modulates ventilation and may have a protective effect against apnea [38,39], potentially contributing to the lesser prevalence, lesser AHI, less severe oxygen desaturation, and shorter apneic episodes exhibited by women.

Women and men with OSA tend to present with different comorbidities, many of which are especially relevant in the context of stroke. Women with OSA have a greater incidence of hypothyroidism and are more likely to experience depression as a result of their OSA [40]. The contribution of OSA to hypertension has been demonstrated to be greater in men [41], and men with OSA may have a higher risk of cardiovascular disease than women [29,42].

Premenopausal women seem to be less susceptible to oxidative stress, one of the central mechanisms by which OSA may elevate stroke risk and hinder recovery [43], as indicated by lower levels of oxidative stress biomarkers and higher antioxidant potential than men. Various studies examining differences in pro-oxidant and antioxidant enzyme activity in men versus women found no difference or yielded inconsistent results [44-50]. Therefore, the sex difference in levels of oxidative stress may be due, at least in part, to the antioxidant capabilities of estrogen. A study by Gaines et al. found the OSA-induced systemic inflammatory response to be more pronounced in males [51].

In this retrospective study, we aim to evaluate the impact of sex on the extent to which OSA affects 90-day functional outcomes following ischemic stroke, as measured by the modified Rankin Scale (mRS) score. Furthermore, we aim to discern whether any sex differences in 90-day functional outcomes among OSA patients are more pronounced within a specific etiology of AIS, as defined by the Trial of Org 10172 in Acute Stroke Treatment (TOAST) classification of ischemic stroke [52].

## Materials and methods

We performed a retrospective analysis of patients with OSA and AIS from the Houston Methodist Hospital Outcomes-Based Prospective Endpoints in Stroke (HOPES) Registry from 2016 to 2022 [53]. The HOPES Registry offers information regarding patient age, sex, race, relevant comorbid conditions, and 90-day outcomes. Patients with documented AIS but without a 90-day mRS score recorded in the registry were excluded. Patients with charts that noted a diagnosis of OSA before the stroke or within the 90 days following their stroke were included in this study. Of 3,887 patients documented with AIS, 291 females and 449 males were found to have OSA within the first 90 days following their ischemic stroke. Charts that noted a relevant diagnosis of OSA were then reviewed for stroke etiology subtype using the TOAST classification. Of these patients with relevant diagnoses of OSA, 185 patients (60 female, 125 male) presented with large-vessel atherosclerosis etiology, 261 (95 female, 166 male) presented with a cardioembolic source of stroke, 96 (44 female, 52 male) presented with small-vessel occlusion, 27 (seven female, 20 male) presented with a stroke of other determined etiology, and 171 (85 female, 86 male) presented with a stroke of undetermined etiology. We have reported descriptive characteristics with means and standard deviations (SDs) for normally distributed continuous variables and medians with interquartile range (IQR) for non-normally distributed continuous variables. Student’s t-test was performed to identify the mean difference between the normally distributed continuous variables, and the Wilcoxon rank sum test was performed to report the median differences between non-normally distributed continuous variables. The chi-square (χ^2^) test was used to identify the differences between categorical variables. The outcome variable was categorized using the mRS, with mRS between 0 and 2 considered a good functional outcome, while mRS 3 to 6 considered a poor functional outcome at 90 days. The independent variable was patient gender. A multivariate logistic regression model was constructed by adjusting for demographics, first admit National Institutes of Health Stroke Scale (NIHSS), and comorbidities on the binary outcome. The odds ratios (ORs) and 95% confidence intervals (CIs) were reported, providing likelihood estimates of a shift to a higher mRS score for a given comparison between females (reference category) and males. Statistical significance was defined as two-tailed p-values <0.05 for all tests. All analyses were performed using R statistical software version 4.1.1 (R Foundation for Statistical Computing, Vienna, Austria).

## Results

From the HOPES registry, a total number of 740 patients were diagnosed with OSA. Of these 740 patients, 291 were females and 449 were males. There was an equal distribution of age(s) in both females (mean = 66.91) and males (mean = 66.60 years) (p = 0.60). Both the female and the male cohorts were predominantly white (53% and 68%, respectively). The female cohort, however, had a higher proportion of black individuals (44% vs. 28%, p < 0.001).

Males had a higher proportion of comorbid conditions such as atrial fibrillation (15% vs. 9%, p = 0.014) and intracranial hemorrhage (6% vs. 2%, p = 0.020) compared to females. Females and males had equal proportions of comorbid conditions such as congestive heart failure (12% vs. 15%, p = 0.23), transient ischemic attack (5% vs. 2%, p = 0.097), hypertension (23% vs. 27%, p = 0.30), end-stage renal disease (6% vs. 10%, p = 0.079), diabetes (15% vs. 16%, p = 0.92), dementia (3% vs. 4%, p = 0.67), coronary artery disease (11% vs. 17%, p = 0.055), and hyperlipidemia (21% vs. 25%, p = 0.33). Incidence of previous stroke was high among both the females and males included in this study (24% and 27%, p = 0.30).

The most prevalent stroke etiology among both females and males was cardioembolism: 95 females and 166 males were identified as having strokes of a cardioembolic origin. Females and males had equal proportions of cardioembolic strokes (33% vs. 37%, p = 0.24) and small vessel (15% vs. 12%, p = 0.18) etiologies. Males, however, had a higher proportion of large artery atherosclerotic (28% vs. 21%, p = 0.03) stroke etiology. Table 1 describes the general characteristics of the study sample.

**Table 1:** Sample characteristics of sleep apnea patients by gender. TOAST = Trial of Org 10172 in Acute Stroke Treatment; TICI = thrombolysis in cerebral infarction; LKW = last known well; NIHSS = National Institutes of Health Stroke Scale; mRS = modified Rankin scale

	Females (N = 291)	Males (N = 449)	P-value
Age, year (mean, SD)	66.91 (±13.69)	66.60 (±12.19)	0.60
Race (N, %)			
White	142 (52.99)	288 (68.09)	<0.001
Black	119 (44.40)	120 (28.37)	<0.001
Asian	7 (2.61)	15 (3.55)	0.66
Ethnicity (N, %)			
Hispanics	25 (9.09)	45 (10.47)	0.61
Non-Hispanics	250 (90.91)	385 (89.53)	0.61
Comorbidities (N, %)			
Congestive heart failure	35 (12.03)	69 (15.37)	0.23
Transient ischemic attack	14 (4.81)	11 (2.45)	0.097
Atrial fibrillation	27 (9.28)	70 (15.59)	0.014
Intracerebral hemorrhage	7 (2.41)	28 (6.24)	0.020
Hypertension	67 (23.02)	119 (26.50)	0.30
End-stage renal disease	18 (6.19)	45 (10.02)	0.079
Diabetes	45 (15.46)	72 (16.04)	0.92
Dementia	8 (2.75)	16 (3.56)	0.67
Coronary artery disease	33 (11.34)	75 (16.70)	0.055
Stroke	69 (23.71)	123 (27.39)	0.30
Hyperlipidemia	62 (21.31)	110 (24.50)	0.33
TOAST Categories (N, %)			
TOAST - Large artery atherosclerosis	60 (20.62)	125 (27.84)	0.030
TOAST - Cardioembolism	95 (32.65)	166 (36.97)	0.24
TOAST - Small vessel	44 (15.12)	52 (11.58)	0.18
TOAST - Other determined etiology	7 (2.41)	20 (4.45)	0.16
TOAST - Undetermined etiology	85 (29.21)	86 (19.15)	0.002
TICI Scores (N, %)			
TICI-2b	6 (20.69)	19 (23.75)	0.80
TICI-2c	2 (6.90)	9 (11.25)	0.72
TICI-3	21 (72.41)	52 (65.00)	0.50
LKW-puncture minutes (median, IQR)	245.00 (194.00–435.00)	290.00 (220.50–611.00)	0.16
First NIHSS (median, IQR)	7.00 (3.00–14.00)	9.00 (4.00–18.00)	0.088
90-day mRS binary classification (N, %)			
mRS <=2	159 (54.64)	229 (51.00)	0.37
mRS 3–6	132 (45.36)	220 (49.00)	0.37
90-day mRS ordinal scale (N, %)			
mRs-0	69 (23.71)	77 (17.15)	0.030
mRs-1	56 (19.24)	79 (17.59)	0.63
mRs-2	34 (11.68)	73 (16.26)	0.088
mRs-3	53 (18.21)	75 (16.70)	0.62
mRs-4	32 (11.00)	65 (14.48)	0.18
mRs-5	23 (7.90)	34 (7.57)	0.89
mRs-6	24 (8.25)	46 (10.24)	0.44

A multivariate logistic regression model after adjusting for demographics, first admit NIHSS, and comorbidities showed that males were at a two times higher risk for developing poor functional outcomes at 90 days (OR = 2.35, 95% CI = 1.06-5.19). Table 2 describes the multivariate logistic regression model.

**Table 2:** Multivariate logistic regression model. NIHSS = National Institutes of Health Stroke Scale

Variables	Odds ratio (95% CI)	P-value
Gender Female	Reference	Reference
Male	2.35 (1.06–5.19)	0.04
Age	1.02 (0.99–1.06)	0.18
Race White	Reference	Reference
Black	1.69 (0.65–4.42)	0.28
Asian	1.67 (0.05–55.77)	0.77
Ethnicity non-Hispanics (Hispanic = Reference)	1.25 (0.3–5.3)	0.76
Congestive heart failure (Yes, No = Reference)	2.14 (0.8–5.68)	0.13
Transient ischemic attack (Yes, No = Reference)	1.09 (0.34–3.45)	0.88
Atrial fibrillation (Yes, No = Reference)	1.04 (0.45–2.38)	0.93
Intracerebral hemorrhage (Yes, No = Reference)	2.02 (0.67–6.1)	0.21
Hypertension (Yes, No = Reference)	0.95 (0.11–8.38)	0.96
End-stage renal disease (Yes, No = Reference)	1.21 (0.48–3.01)	0.69
Diabetes (Yes, No = Reference)	1.59 (0.69–3.66)	0.27
Dementia (Yes, No = Reference)	1.19 (0.38–3.71)	0.76
Coronary artery disease (Yes, No = Reference)	0.31 (0.11–0.85)	0.02
Hyperlipidemia (Yes, No = Reference)	0.85 (0.25–2.86)	0.8
First NIHSS	1.15 (1.08–1.22)	<0.001

## Discussion

In our retrospective analysis of patients presenting with OSA, males were found to have over two times higher risk of poor functional outcomes at 90 days, as measured by the mRS score. The HOPES Registry included 291 females and 449 males diagnosed with OSA within the 90 days following their AIS, accounting for 15% of females and 23% of males documented with AIS in the registry. Given the considerable prevalence of OSA among patients recovering from AIS, especially in males, the results of this study indicate that greater emphasis on early diagnosis and treatment of OSA may be necessary to reduce the disproportionate incidence of poor functional outcomes among apneic patients in general, but especially among apneic males.

The sex differences revealed by this retrospective study offer insights into the most significant mechanisms by which OSA likely impedes functional recovery from stroke and factors that may reduce the negative impact of OSA on recovery. Cardioembolic and large artery atherosclerotic etiologies were the most commonly determined etiologies among both sexes in this cohort. Given that cardioembolic stroke was by far the most common etiology of stroke in this study, the negative intrathoracic pressure swings, increased cardiac oxygen demand, and oxidative stress induced by OSA may be of particular concern in the context of stroke recovery and may explain the sex gap in functional recovery that we observed. Men are more susceptible to oxidative damage than women [43], which may be a key mechanism preventing effective neurological recovery and management of cardiac risk factors after cardioembolic stroke. It is possible that apneic males may experience more drastic and damaging negative pressure swings as they experience a greater frequency of full airway obstructions, longer episodes of obstruction, and greater ventilatory demand relative to their female counterparts [31,35]. The greater ventilatory demand, more severe oxygen desaturations, and greater incidence of hypertension among males with OSA may disproportionately increase their cardiac oxygen demand, exacerbating the immense stress put on the atrial walls via drastic pressure swings [27,28,41].

The prevalence of the large artery atherosclerotic stroke subtype in this cohort further supports the roles of the severity of oxygen desaturations and susceptibility to oxidative damage in determining the incidence of poor outcomes following ischemic injury. A study by Schulz et al. found that the contribution of OSA to large artery atherosclerosis is related to the severity of oxygen desaturation [54], which is typically greater in apneic men. Because oxidative stress and systemic inflammation play a central role in damaging the endothelium of the large arteries during apneic events, the increased susceptibility to oxidative stress and the more pronounced inflammatory response observed in males with OSA may predispose them to greater atherosclerotic lesion progress and stenosis of the large arteries, impairing effective perfusion to the affected tissues of the brain.

Given that only a fraction of patients within the HOPES Registry underwent screening for OSA, there may exist a considerable population of patients with undocumented OSA in the registry which could not be included in this study. Future prospective studies that avoid this selection bias are needed. Another limitation of this study is the lack of pre-admission mRS scores for comparison. Because 24% of females and 27% of males had a prior diagnosis of stroke, pre-admission mRS scores are necessary to account for cases in which mRS scores indicating poor outcomes already exist from the prior stroke. Furthermore, the males included in this study had a higher proportion of comorbidities such as atrial fibrillation and intracranial hemorrhage compared to their female counterparts, which may have had a role in the poor 90-day outcomes noted among the males.

A particularly significant limitation of this study was that patients with both treated and untreated OSA were included. Compliance with continuous positive airway pressure (CPAP) treatment for OSA could not be definitively determined based on chart review for the apneic patients included in this study. Future studies are needed to determine if sex differences in functional recovery may be effectively reduced among stroke survivors compliant with early CPAP treatment. Polysomnography should be performed early during the post-stroke period, especially among males, to screen for OSA so that treatment may be implemented as early as possible. Treatment with CPAP should be strongly encouraged for those who present with OSA to reduce symptoms and the likelihood of poor functional outcomes. There is evidence that treatment of OSA with CPAP in stroke survivors accelerates neurological recovery, improves functional and motor outcomes, lowers blood pressure, restores nocturnal *dipping*, reduces mortality, protects against new vascular events, and mitigates depressive symptoms [55-61]. Several studies on CPAP use among apneic stroke survivors have reported disproportionately poor adherence within this population [56,62]. Therefore, further research is warranted to investigate alternative methods of OSA treatment for stroke survivors and effective interventions to improve CPAP adherence among stroke survivors.

## Conclusions

In our study of AIS patients with OSA, the male sex was associated with a higher risk of poor 90-day functional outcome after AIS, especially of large artery atherosclerotic etiology, after adjusting for comorbidities and demographics. Earlier discovery of OSA and adherence to CPAP treatment may help reduce the risk of poor functional outcomes among apneic male stroke survivors.
